# Supplementary material for: Oligomalt, a New Slowly Digestible Carbohydrate, Is Well Tolerated in Healthy Young Men and Women at Intakes Up to 180 Gram per Day: A Randomized, Double-Blind, Crossover Trial
Source: Nutrients. 2023 Jun 15;15(12):2752. doi: 10.3390/nu15122752 (PMC10305601; doi:10.3390/nu15122752)
Supplement: Supplementary file 1 [file nutrients-15-02752-s001.zip › nutrients-2425818-supplementary.pdf]

Supplementary Materials

Table S1: Full list of inclusion and exclusion criteria

Inclusion criteria	• Male or female participant<br>• Healthy status<br>• Age between 18 and 65 years<br>• Body mass index (BMI) between 18.5 and 29.9 kg/m^2<br>• Able to understand and sign informed consent form.<br>• Having a smartphone with android or iOS version compatible with Patient Cloud application
Exclusion criteria	• Fasting plasma glucose ≥ 6.1mmol/L at screening<br>• Type 1 or type 2 diabetes<br>• Pregnant or lactating women<br>• Known food allergy or intolerance to test product<br>• Treatment with anorectic drugs, glucose-lowering drugs, steroids, medications known to affect glucose metabolism and/or gastric motility, or any condition known to affect gastro-intestinal integrity and food absorption<br>• Colonoscopy, irrigoscopy or other bowel cleansing procedures four weeks prior the test<br>• Any concomitant medication potentially interfering with study procedures and assessment (antibiotics, antiacids) or other medications impacting transit time<br>• Major medical/surgical event in the last 3 months potentially interfering with study procedures and assessments<br>• Volunteer who cannot be expected to comply with the protocol<br>• Abnormal bowel transit, history of a gastrointestinal disorder (e.g., inflammatory bowel disease, diverticular diseases, colon cancer), or history of chronic constipation with passage of fewer than 3 spontaneous bowel movements per week on average or chronic or recurrent diarrhea with spontaneous bowel movements more often than 3 times daily<br>• Recent episodes of acute gastrointestinal illness<br>• Habitual consumption of more than four servings per day of high-fiber food or extreme dietary<br>• Ongoing or recent weight loss interventions<br>• Alcohol intake higher than 2 servings per day A serving is 0.4 dl of strong alcohols, 1 dl of red or white wine, or 3 dl of beer<br>• Score of “severe” symptom for any of the symptoms included in the gastrointestinal symptom questionnaire<br>• Family or hierarchical relationships with Clinical Innovation Lab team

Figure S1: Schematic illustration of study scheme

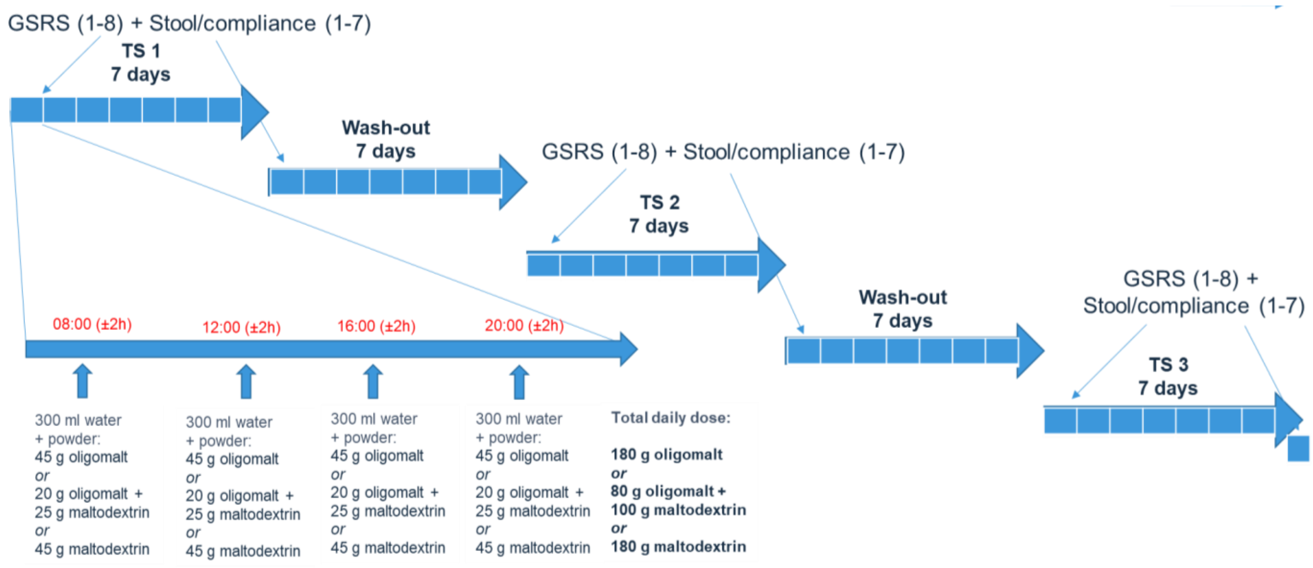

Abbreviations: GSRS – Gastrointestinal Symptom Rating Scale; TS – intervention sequence
